# Robot-Assisted Lobectomy for Lung Cancer Complicated by Lymphangioleiomyomatosis

**DOI:** 10.70352/scrj.cr.26-0211

**Published:** 2026-06-27

**Authors:** Harushi Ueno, Yuta Kawasumi, Heng Huang, Taiki Ryo, Yoshito Imamura, Tatsuya Masuda, Yasuhisa Ichikawa, Minoru Sugihara, Hirofumi Takenaka, Hiroki Watanabe, Keita Nakanishi, Yuka Kadomatsu, Taketo Kato, Shota Nakamura, Tetsuya Mizuno, Toyofumi Fengshi Chen-Yoshikawa

**Affiliations:** Department of Thoracic Surgery, Nagoya University Graduate School of Medicine, Nagoya, Aichi, Japan

**Keywords:** lymphangioleiomyomatosis (LAM), lung cancer, robot-assisted thoracoscopic surgery (RATS), case report, intraoperative air leak

## Abstract

**INTRODUCTION:**

Lymphangioleiomyomatosis (LAM) is a rare systemic disease characterized by progressive cystic destruction of the lung parenchyma, resulting in extreme parenchymal fragility. Surgical treatment of lung cancer in patients with LAM is technically challenging due to the high risk of intractable air leaks. We report a case of robot-assisted thoracoscopic surgery (RATS) for lung cancer in a patient with LAM, highlighting a strategic hybrid approach to managing the exceptionally fragile lung tissue.

**CASE PRESENTATION:**

A 56-year-old woman with tuberous sclerosis complex-associated LAM was diagnosed with Stage IA1 adenocarcinoma in the left upper lobe. We performed a RATS left upper lobectomy using the da Vinci Xi system (Intuitive Surgical, Sunnyvale, CA, USA). To protect the exceptionally fragile lung, a “no-touch” retraction technique was employed using rolled gauze and blunt-tipped robotic instruments. A fissureless technique was applied to minimize parenchymal injury. Crucially, for the division of the incomplete interlobar fissure and the bronchus, we utilized manual staplers equipped with bioabsorbable reinforcement material (buttressed staplers) deployed by a bedside assistant, which provided superior sealing for the fragile lung compared to the robotic staplers available at that time. Despite these precautions, a pinhole air leak occurred in the S8 segment, likely due to unintentional contact with a robotic joint, illustrating the extreme sensitivity of the LAM lung. This was identified via a meticulous sealing test and repaired with polyglycolic acid sheets and fibrin glue. The patient was discharged on POD 5 without persistent air leaks. At 1 year post-surgery, her respiratory function was well-preserved, exceeding predicted values.

**CONCLUSIONS:**

RATS offers superior visualization for lung cancer surgery in patients with LAM. However, given the extreme fragility of the lung, the selective use of manual buttressed staplers is a vital adjunct to prevent postoperative air leaks. Optimal outcomes depend on a constant awareness of lung fragility, a hybrid technical strategy, and a rigorous intraoperative sealing test to identify and repair even minor pleural injuries.

## Abbreviations

ICS
: intercostal space
LAM
: lymphangioleiomyomatosis
RATS
: robot-assisted thoracoscopic surgery

## INTRODUCTION

LAM is a rare systemic disease characterized by the abnormal proliferation of atypical smooth muscle-like cells, known as LAM cells. In the lungs, it particularly causes diffuse thin-walled cysts, leading to recurrent pneumothorax and respiratory dysfunction.^1,2)^

While the coexistence of lung cancer in patients with LAM is rare, its surgical treatment presents significant technical challenges, such as intractable intraoperative and postoperative air leaks due to the fragile lung parenchyma and incomplete interlobar fissures. In recent years, RATS has become widespread in the field of lung cancer surgery, and its high-definition 3D imaging and multi-jointed instruments, which allow for precise manipulation, have been shown to contribute to safer and less invasive procedures.^3)^

Herein, we report a case of early-stage lung cancer with concomitant LAM, for which we performed RATS. We discuss the utility of RATS and the specific precautions required, along with a review of the relevant literature.

## CASE PRESENTATION

**Fig. 1:**
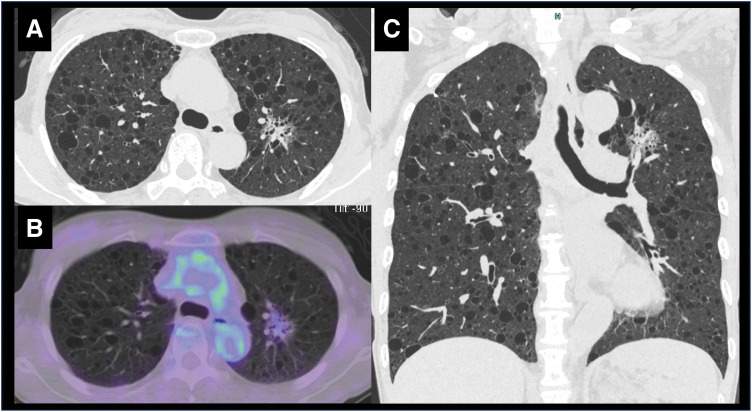
(**A**) A chest CT scan shows an enlarging part-solid ground-glass opacity within a lung that has numerous thin-walled cysts. (**B**) PET-CT revealed radiotracer uptake in the nodule, with an SUVmax of 1.39. (**C**) Considering the tumor’s location, lobectomy was selected as the surgical procedure. SUVmax, maximum standardized uptake value

The patient was a 56-year-old woman with a history of tuberous sclerosis complex and LAM who was undergoing regular surveillance. She was a former smoker (12 pack-years) who had quit at age 32. A ground-glass nodule in her left upper lobe, which had been noted for 2 years, was observed to have increased in size (**Fig. 1A**). Bronchoscopy was performed, which led to a diagnosis of adenocarcinoma. Preoperative pulmonary function tests revealed an obstructive ventilatory impairment, with the following values: forced vital capacity (FVC) 2630 mL (%FVC 101.9), forced expiratory volume in 1 second (FEV_1_) 1820 mL (%FEV_1_ 85.8), and FEV_1_/FVC ratio (FEV_1_%) 69.2. Based on the results of PET-CT (**Fig. 1B**) and brain MRI, she was diagnosed with left upper lobe lung cancer, cT1aN0M0, Stage IA1. Considering the maximum tumor dimension of 30 mm and its proximity to the hilum in the left upper lobe (**Fig. 1C**), we selected a robot-assisted left upper lobectomy. Although recurrence was considered unlikely, ND2a-2 was performed because the patient’s general condition would allow for postoperative chemotherapy if metastasis were identified. The surgery was performed under general anesthesia in the right lateral decubitus position with 1-lung ventilation. A total of 5 ports were placed. An 8-mm camera port was placed in the 8th ICS on the mid-axillary line, with additional 8 mm ports in the 6th ICS on the anterior axillary line, the 8th ICS on the posterior axillary line, and the 8th ICS on the scapular line. An assistant port was placed in the 8th ICS on the anterior axillary line, and a stable surgical field was maintained by CO_2_ insufflation at 5 mmHg using the AirSeal system (MC Medical, Tokyo, Japan).

**Fig. 2:**
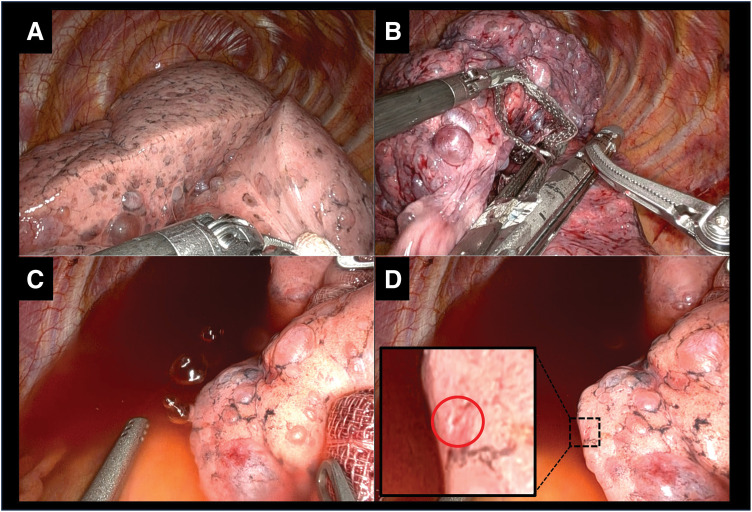
(**A**) View of the left lung showing multiple thin-walled cysts on the surface, characteristic of LAM. (**B**) Division of the interlobar fissure with a buttressed stapler. (**C**) An intraoperative sealing test. After filling the thoracic cavity with saline, air bubbles were seen rising from the S8 segment of the left lung, indicating an air leak. (**D**) A pinhole-sized injury (red circle, magnified view) was identified, considered to be an iatrogenic injury caused by unintentional contact from a robotic instrument. LAM, lymphangioleiomyomatosis

Due to diffuse cystic changes from LAM, the lung had numerous thinned bullae, and the fissure was incomplete (**Fig. 2A**). For lung retraction, the lung was gently manipulated using rolled gauze with blunt-tipped, less traumatic instruments such as the Cadiere forceps and Tip-Up fenestrated grasper, without directly grasping the parenchyma. All vascular, bronchial, and parenchymal divisions were performed using manual staplers by the assistant through the assistant port. First, the left upper lobe was retracted dorsally, and the superior pulmonary vein was secured and divided. Subsequently, dissection around the pulmonary artery was advanced from the dorsal side, and the branches of the pulmonary artery supplying the upper division were sequentially divided. The left upper lobe bronchus was encircled and divided after confirming sufficient aeration in the lower lobe under 2-lung ventilation. Dorsal to the bronchial stump, the main pulmonary artery was exposed, and the branches supplying the lingular division were divided. Finally, the incomplete interlobar fissure was divided from the ventral side using 3 staplers. For the interlobar fissure, a buttressed stapler was chosen to mitigate the risk of air leakage (**Fig. 2B**).

A meticulous water-seal test at a pressure of up to 15 mmHg identified 2 sites of air leakage: a pinhole in the S8 segment of the lower lobe (**Fig. 2C** and **2D**) and the staple line of the interlobar fissure. Both sites were patched with a polyglycolic acid sheet and sealed with fibrin glue. A 24-Fr chest tube was placed, and the incisions were closed. The operative time was 205 min, with a console time of 179 min and a blood loss of 3 mL.

Pathological examination of the resected left upper lobe revealed an invasive adenocarcinoma with a total size of 2.5 × 2.5 cm. Histologically, the tumor was predominantly lepidic (70%) with a papillary component (30%). The maximal invasive component measured 1.0 × 0.7 cm. There was no evidence of pleural, lymphatic, or vascular invasion (pl0, Ly0, V0), and all dissected lymph nodes were negative for metastasis. The final pathological stage was pT1aN0M0, Stage IA1. The tumor was positive for an EGFR L858R mutation. Concurrently, the lung parenchyma showed multiple cysts with destruction of airspaces. The cyst walls contained epithelioid cells consistent with LAM cells, a finding supported by immunohistochemistry (partially positive for HMB-45, weakly positive for PgR).

**Fig. 3:**
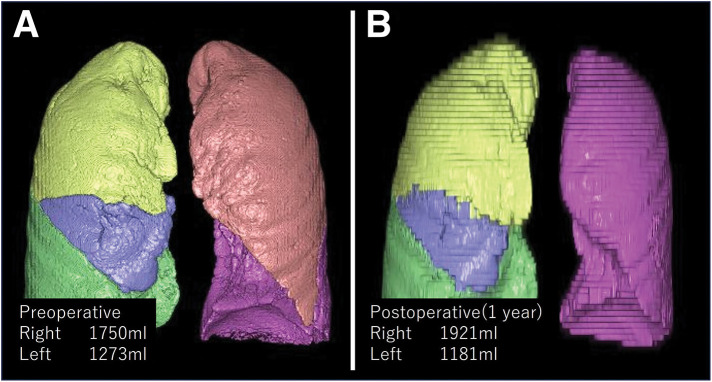
(**A**) Preoperative 3D-CT volumetry. The right and left lung volumes were 1750 and 1273 mL, respectively. (**B**) Postoperative 3D-CT volumetry at 1 year after left upper lobectomy. The total left lung volume decreased to 1181 mL, but the remaining left lower lobe demonstrated significant compensatory hyperinflation, expanding into the space of the resected lobe. The contralateral right lung also showed an increased total volume of 1921 mL.

Postoperatively, there was no persistent air leak; the chest tube was removed on POD 3, and she was discharged without complications on POD 5. A pulmonary function test at 1 year post-surgery showed: FVC 2400 mL (%FVC 94.4%), FEV_1_ 1580 mL (%FEV_1_ 75.9), and FEV_1_% 65.8 (**Table 1**). Comparison of preoperative and 1-year postoperative chest 3D-CT scans showed that the total lung volume remained almost unchanged, while the left lower lobe demonstrated compensatory hyperinflation (**Fig. 3**). As of 1 year and 3 months post-surgery, she has no evidence of recurrence.

**Table 1:** Preoperative and 1-year postoperative pulmonary function test results

Parameter	Preoperative		Postoperative (1 year)	
FVC (mL)	2630	101.9%	2400	94.4%
FEV_1_ (mL)	1820	85.8%	1580	75.9%
FEV_1_% (%)	69.2		65.8	
DLCO (mL/min/mmHg)	9.45	66.1%	9.64	71.7%
DLCO/VA (mL/min/mmHg/L)	2.94	61.3%	4.73	65.3%

Data are presented as measured values, with the percentage of predicted values shown in parentheses.

DLCO, diffusing capacity of the lungs for carbon monoxide; DLCO/VA, DLCO/alveolar volume; FVC, forced vital capacity; FEV_1_, forced expiratory volume in 1 second; FEV_1_%, FEV_1_/FVC ratio

## DISCUSSION

This case highlights the surgical management of lung cancer coexisting with LAM, a rare combination found in only 2.2% of patients with LAM, according to a large Japanese cohort.^4)^ Given the patient’s adequate pulmonary reserve and the fact that she would reach the eligibility age limit for lung transplantation (60 years) following a 5-year observation period, surgical intervention for lung cancer was deemed the most appropriate clinical priority. To our knowledge, this is the first report of a RATS approach for this condition. The lung parenchyma in LAM is exceptionally fragile and prone to intractable air leaks.^5)^ We previously reported that RATS may carry a higher risk for prolonged air leaks compared to video-assisted thoracoscopic surgery in routine cases,^6)^ a risk that is significantly magnified in LAM. In this case, a hybrid approach was necessary; manual buttressed staplers were used because robotic staplers with integrated reinforcement were unavailable at that time. Deguchi et al. demonstrated that buttressed staples are useful for minimizing the incidence of postoperative air leakage.^7)^ Despite a fissureless technique and buttressed stapling, air leaks occurred. Notably, the pinhole injury in the S8 segment—an area outside the primary operative field—was likely caused by unintentional contact with a robotic joint. This underscores the exquisite fragility of the LAM lung, where even minor iatrogenic contact can cause damage. Therefore, surgeons must perform an exhaustive sealing test, inspecting not only the staple lines but also areas where accidental instrument contact may have occurred.

From a respiratory function standpoint, this patient had a pre-existing obstructive ventilatory impairment. The predicted postoperative FVC and FEV_1_ after a left upper lobectomy (4 segments) were calculated to be about FVC 2040 mL and FEV_1_ 1410 mL using a simple segment-counting method (preoperative FVC or FEV_1_ × 14/18). However, the actual measured values at 1 year post-surgery were FVC 2400 mL and FEV_1_ 1580 mL, which were well-maintained and exceeded the prediction. It has been shown in many reports that actual postoperative lung function is often better than that predicted by simple calculation formulas,^8)^ and it is thought that a similar trend can be observed in patients with LAM. Consistent with this, the total lung volume calculated by 3D-CT showed little change between the preoperative period and 1 year post-surgery. This was attributed to compensatory changes in both lungs, especially the hyperinflation of the remaining left lower lobe.

## CONCLUSIONS

RATS is a feasible and effective approach for lung cancer in patients with LAM, offering superior visualization. However, success is contingent upon a constant awareness of parenchymal fragility, the selective use of buttressed staplers, and a rigorous intraoperative sealing test to ensure a definitive repair of any pleural injuries.

## DECLARATIONS

### Availability of data and materials

The data supporting the findings of this study are available from the corresponding author, Harushi Ueno, upon request.
